# Case Report: Early-onset or recalcitrant cytopenias as presenting manifestations of activated PI3Kδ syndrome

**DOI:** 10.3389/fped.2024.1494945

**Published:** 2024-11-27

**Authors:** Allison S. Remiker, Joao Pedro Matias Lopes, Rohith Jesudas, Alexandra Superdock, Nami Park, Irina Pateva

**Affiliations:** ^1^ Department of Pediatrics, Medical College of Wisconsin, Milwaukee, WI, United States; ^2^ Division of Hematology/Oncology/Blood and Bone Marrow Transplantation, Children's Wisconsin Hospital, Milwaukee, WI, United States; ^3^ Division of Pediatric Allergy/Immunology, UH Rainbow Babies & Children's Hospital, Cleveland, OH, United States; ^4^ Department of Hematology, St. Jude Children's Research Hospital, Memphis, TN, United States; ^5^ Medical Affairs, Pharming Healthcare, Inc., Warren, NJ, United States; ^6^ Department of Pediatrics, Case Western Reserve University, Cleveland, OH, United States; ^7^ Hematologic Malignancies II, US Food and Drug Administration, Silver Spring, MD, United States

**Keywords:** recalcitrant cytopenias, early-onset cytopenias, inborn error of immunity, activated PI3Kδ syndrome, case series, genetic testing

## Abstract

**Background:**

Patients with recurrent, chronic, or refractory cytopenias represent a challenging subgroup that may harbor an underlying diagnosis, such as an inborn error of immunity (IEI). Patients with IEIs such as activated phosphoinositide 3-kinase delta syndrome (APDS), frequently have hematologic manifestations, but these are not often reported as presenting symptoms. As a result, IEIs may be overlooked in patients presenting with early and/or recalcitrant cytopenias. Here, we describe the diagnostic journey and management of three patients who presented to a pediatric hematologist/oncologist with early-onset or recalcitrant cytopenias and were ultimately diagnosed with APDS.

**Case presentations:**

Patients presented with early-onset and/or refractory cytopenias, with two of the three developing multilineage cytopenias. Prior to an APDS diagnosis, two patients underwent a total of approximately 20 procedures, including biopsies, invasive endoscopies, and imaging, with one undergoing eight differential diagnoses that were ruled out through additional testing. Recalcitrant cytopenias, a history of infection, and a family history of lymphoproliferation, infection, or autoimmunity raised suspicion of an underlying IEI, leading to genetic testing. Genetic testing identified a pathogenic variant of *PIK3CD* in each patient, resulting in the diagnosis of APDS. Following these diagnoses, two patients underwent modifications in the management of care with the administration of intravenous immunoglobulin therapy (IVIG), the mTOR inhibitor sirolimus, or surgical procedures. These treatment modifications either improved or resolved the cytopenias. The third patient showed improvement in immune thrombocytopenia with IVIG 1 month prior to receiving a definitive diagnosis. Following diagnosis, follow-up genetic testing of family members led to the identification of additional cases of APDS.

**Conclusions:**

These cases highlight the importance of early genetic evaluation in patients with early-onset or recalcitrant cytopenias and demonstrate the challenges of differential diagnosis. In addition, these cases demonstrate beneficial changes in management and outcomes that can follow a definitive diagnosis, including the identification of targeted treatment options. Collectively, this case series supports the notion that underlying IEIs should be considered in the workup of early-onset or recalcitrant cytopenias, particularly in patients who present with a combination of hematologic and immunologic manifestations that are refractory to treatment, manifest at an unusually young age, or can be tied to family history.

## Introduction

The majority of children with autoimmune cytopenias experience mild symptoms that resolve spontaneously. However, a subgroup of patients who present with chronic or multilineage cytopenias may have underlying conditions, such as inborn errors of immunity (IEIs) (1, 2). Patients with IEIs, such as activated phosphoinositide 3-kinase delta syndrome (APDS), frequently have with hematologic manifestations that are not often reported as presenting symptoms (3–7). As a result, IEIs, such as APDS, may be overlooked in patients who present with early-onset and/or recalcitrant cytopenias.

APDS manifestations are driven by pathogenic gain-of-function variants in *PIK3CD* (APDS1) or loss-of-function variants in *PIK3R1* (APDS2), causing hyperactive phosphoinositide 3-kinase delta signaling (3–9). Phosphoinositide 3-kinase delta hyperactivation alters lymphocyte development and function, resulting in combined immune dysregulation and deficiency (10–12). Patients with APDS may present with manifestations early in life, with infections occurring before 1 year of age(13). Respiratory infections are often followed by lymphoproliferation, but patients may also experience gastrointestinal (GI) complications, autoimmune cytopenias, and lymphoma (4). Elevated immunoglobulin M (IgM) levels are commonly reported in patients with APDS, leading to an initial diagnosis of hyper-IgM syndrome in some. Bronchiectasis is more common in APDS1, while failure to thrive and neurologic disorders, including neurodevelopmental delay, learning disabilities, autism spectrum disorder, seizures, anxiety, and depression, are more frequently reported in APDS2 (4, 10, 11, 13, 14). Despite differences in the incidence of certain manifestations, hematologic complications have been reported in patients with APDS1 and APDS2 (4).

While case reports on hematologic manifestations in patients with APDS exist, they have focused largely on the development of malignancy (15–18). Although patients with APDS are at an increased risk for lymphoma, recalcitrant cytopenias may be an early warning sign (1, 10, 11). Therefore, we sought to highlight the diagnostic journey and management of three patients who presented to a pediatric hematologist/oncologist with early-onset or recalcitrant cytopenias to highlight the challenges in APDS diagnosis and the importance of genetic evaluation.

## Case presentation

### Patient 1

**Figure 1:**
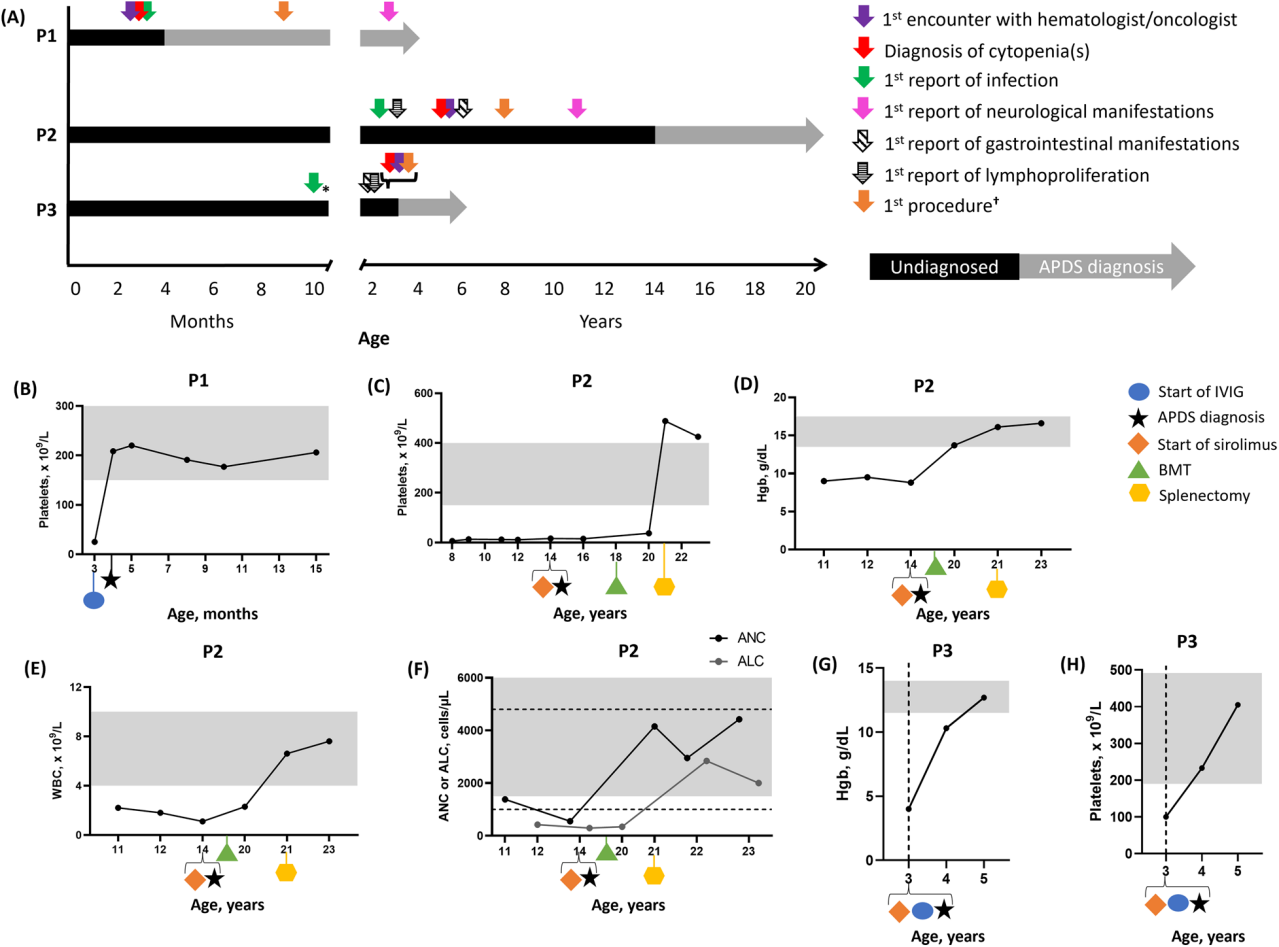
Patient vignette timelines and hematologic parameters over time. **(A)** Timelines for P1, P2, and P3. **(B)** P1 platelet levels. Three values were averaged to plot data at 4 months old. P2 platelet **(C)**, Hgb **(D)**, WBC **(E)**, and ANC/ALC levels **(F)**. P3 Hgb **(G)** and platelet **(H)** levels. In **(B–E)** and **(G,H)**, the gray boxes indicate normal ranges. In **(F)**, the gray box indicates the normal range for ANC and the dashed lines indicate the normal range for ALC. *Medical records indicated that infections started at a young age. †Procedures included imaging (e.g., ultrasound or x-ray) or biopsy (e.g., bone marrow). ALC, absolute lymphocyte count; ANC, absolute neutrophil count; APDS, activated PI3Kδ syndrome; BMT, bone marrow transplant; Hgb, hemoglobin; IVIG, intravenous immunoglobulin replacement therapy; P1, patient 1; P2, patient 2; P3, patient 3; WBC, white blood cell.

Patient 1 (P1) is a 3-year-old White boy who was first admitted for inpatient service at a pediatric hematology clinic for severe thrombocytopenia at 3 months of age (Figure 1A).

Initial examination revealed a parainfluenza/rhinovirus infection, a petechial rash at a tourniquet site and on both cheeks and a maculopapular rash on the upper chest and clavicular areas, with the latter resolving within weeks. His platelet count was low (25 × 10^9^/L; normal range, 150–450 × 10^9^/L), but his platelet volume was within the normal range (901 fl; normal range, 7.4–10.4 fl). Based on these findings, immune thrombocytopenia purpura (ITP) was suspected, and he received a single dose of intravenous immunoglobulin (IVIG; 1 g/kg) replacement therapy, which was well tolerated (Figure 1B). From 3 to 15 months of age, his hemoglobin (Hgb) level, white blood cell (WBC) count, absolute neutrophil count (ANC), and absolute lymphocyte count (ALC) were regularly monitored and remained within normal ranges (Supplementary Figure S1).

At a 1-month follow-up, platelet-associated autoantibodies were detected against glycoprotein IIB/IIIA, IA/IIA, IV, and human leukocyte antigen class I. At this time, the ITP had resolved with IVIG therapy. Family history revealed a history of recurrent sinopulmonary infections and an IEI, known as common variable immunodeficiency (CVID), in P1's paternal aunt. Together with the onset of ITP at a young age, these findings prompted the suspicion of an underlying IEI. Immune profiling of the T-cell compartment revealed low counts of naïve CD4^+^ and CD8^+^ T cells, with elevated effector memory CD8^+^ T cells, senescent CD4^+^ T cells, and the CD4:CD8 T-cell ratio. Effector memory CD4^+^ and senescent CD8^+^ T cells were within normal limits (WNLs). Total B cells were elevated (Table 1). The results from P1's immune workup and family history contributed to the decision to complete genetic testing, which revealed a pathogenic variant in *PIK3CD* (p.Glu1021Lys), confirming an APDS1 diagnosis.

**Table 1:** Immune profiling of patient 1.

Immune subset/immunoglobulin	Age 3 months	Age 4 months	Age 8 months	Age 10 months	Age 15 months	Age 3 years
Naïve CD4^+^ T cells, %	—	↓, *27.76* (61.9–89.1)	—	—	—	↓, *24.81* (28–47)
Naïve CD8^+^ T cells, %	—	↓, *22.92* (47.1–97.3)	–	–	–	16.91 (16–30)
Effector memory CD4^+^ T cells, %	—	4.99 (0.43–5.53)	—	—	—	↑, *12.00* (0.4–3.4)^a^
Effector memory CD8^+^ T cells, %	—	↑, *31.76* (0.37–10.32)	—	—	—	↑, *17.00* (0.3–12.7)^a^
Senescent CD4^+^ T cells, %	—	↑, *32.82* (2.73–14.5)	—	—	—	—
Senescent CD8^+^ T cells, %	—	1.45 (0.11–2.56)	—	—	—	—
CD4:CD8 T-cell ratio	—	↑, *4.28* (1.18–3.89)	—	—	—	1.47 (0.9–3.0)^a^
Total B cells, cells/µl	↑, *4,645* (745–3,499)	↑, *3,596* (745–3,499)	—	1,767 (835–3,774)	—	1,027 (390–1,400)
Naïve B cells, %	—	—	86.93 (86.74–98.34)	—	—	↑, *80.80* (49.7–77.1)^a^
Transitional B cells, %	—	—	0.07 (0–5.09)	—	—	–
Plasmablasts, %	—	—	0.22 (0.05–3.66)	—	—	–
Switched memory B cells, %	—	—	0.56 (0.01–4.71)	—	—	2.49 (1.5–4.1)^b^
Non-switched memory B cells, %	—	—	8.14 (0.30–8.17)	—	—	↓, *4.52* (4.8–16.1)^a^
IgG, mg/dl	—	—	429 (208–868)	377 (282–1,026)	651 (331–1,165)	↑, *1,070* (342–913)
IgA, mg/dl	—	—	16 (11–89)	—	28 (14–105)	↑, *222* (10–118)
IgM, mg/dl	—	—	↑, *238* (32–120)	—	↑, *215* (41–164)	↑, *214* (30–129)

↑, above normal limits; ↓, below normal limits.

^a^ Reference range from van Gent (35).

^b^ Reference range from Morbach (36).

Normal range values are shown in parentheses. Values outside the normal range are italicized. Normal ranges were provided by the healthcare provider or from the literature (35, 36).

Following the APDS1 diagnosis, Ig and lymphocyte levels were monitored, as these are often altered in APDS (10, 11). At 8 months of age, his IgM was elevated, while IgG and IgA levels were WNLs. Analysis of the B-cell compartment revealed normal levels of naïve, transitional, plasmablasts, and switched and non-switched memory B cells. Hepatosplenomegaly was absent during an abdominal ultrasound at 9 months of age.

Due to early diagnosis, P1 is monitored by appropriate specialists to prevent progressive outcomes. At 3 years of age, he no longer required hematology follow-up, as his ITP had resolved. He now visits immunology every 6 months to monitor an increase in infections that started during preschool. Naïve CD4^+^ T cells remained low, while naïve CD8^+^ T cells reached the normal range. Effector memory T cells were elevated, but the CD4:CD8 T-cell ratio normalized. Naïve B cells were elevated, while non-switched memory B cells were low. Total B cells decreased WNLs. IgM, IgG, and IgA were elevated. In addition, P1 developed asthma, which was also reported in his paternal aunt and mother. Following the development of bronchiectasis, he began pulmonology visits and treatment, which included budesonide/formoterol, vest therapy, albuterol, and azithromycin. In early toddlerhood, his pediatrician diagnosed him with mixed receptive-expressive speech delays requiring an assisted communication device and gross motor delay followed by physical therapy.

Following P1's diagnosis, his sister underwent genetic testing due to a history of mild tonsillar hypertrophy since 19 months of age. She was diagnosed with APDS at 2 years of age and was found to have global developmental delay and asthma, which is monitored by pulmonology.

No adverse events were reported during P1’s treatment.

### Patient 2

Patient 2 (P2) is a 23-year-old White man who was referred to a pediatric hematologist/oncologist by his immunologist for ITP at 5 years of age.

His initial examination with pediatric hematology/oncology revealed lymphadenopathy, petechiae, a fever requiring hospitalization, and splenomegaly. His medical history revealed recurrent sinopulmonary infections (i.e., recurrent otitis and several episodes of pneumonia) since 2 years of age. Recurrent sinopulmonary infections, along with low IgG levels and a poor antibody response, prompted a CVID diagnosis that was treated with IVIG (500 mg/kg Q4 weeks) at 3 years of age. Lymphadenopathy and food allergies were also reported at this time. At 4 years of age, he received trimethoprim/sulfamethoxazole prophylactically. Recurrent infections and lymphoproliferation were reported in his maternal half-brother and half-sister, while food allergy was reported in his half-sister.

At 6 years of age, P2 was diagnosed with eosinophilic esophagitis, followed by asthma 1 year later; both conditions were also present in his half-sister. At 8 years of age, he exhibited symptoms of infectious Epstein-Barr virus (EBV)-related mononucleosis, which, along with lymphadenopathy, prompted suspicion of X-linked lymphoproliferative disease, which was excluded as no pathogenic variants were identified in *SH2D1A*. His platelet levels were low (6 × 10^9^/L; normal range, 150–400 × 10^9^/L), occasionally requiring high-dose IVIG and rituximab (Figure 1C). Due to ITP and CVID, a bone marrow biopsy (BMBX) was performed at 8 years of age, revealing normocellular results (90%) with increased megakaryocytes and eosinophilia. Due to CVID, lymphadenopathy, and ITP, a computed tomography scan of the chest, abdomen, and pelvis was performed, revealing hepatosplenomegaly at 9 years of age.

At 11 years of age, pneumonia (*Haemophilus influenzae* and *Pseudomonas aeruginosa*), seizures, and an autoimmune lymphoproliferative syndrome (ALPS)-like phenotype were reported. A BMBX was performed at this time due to ALPS, lymphadenopathy, and ITP, but it revealed the same results as reported 3 years earlier. P2 experienced anemia (Hgb, 9 g/dl; normal range, 13.5–17.5 g/dl), while ITP persisted (Figure 1D). WBC (2.2 × 10^9^/L; normal range, 4–10 × 10^9^/L), ANC (1,380 cells/µl; normal range, 1,500–10,000 cells/µl), and ALC (420 cells/µl; normal range, 100–4,800 cells/µl) were also low (Figures 1E,F).

Routine monitoring of immunoglobulins at 12 years of age revealed low IgA and IgM levels, with normal IgG levels (Table 2). The CD4:CD8 T-cell ratio was WNLs. A *Candida* infection of the esophagus and tremor were reported at 12 and 13 years of age, respectively. Due to lymphadenopathy and suspected gastroesophageal reflux disease, an esophagogastroduodenoscopy/colonoscopy was performed at 13 years of age, revealing eosinophilic esophagitis and lymphoid hyperplasia of the GI tract. CVID and suspected bronchiectasis led to a computed tomography scan, revealing mild central bronchiectasis. Another BMBX was normocellular (70%) with mild granulocytic and megakaryocytic hyperplasia and benign lymphoid aggregates. An abdominal ultrasound revealed periorbital lymph nodes.

**Table 2:** Immune profiling of patient 2.

Immune subset/immunoglobulin	Age 12 years	Age 14 years	Age 15 years	Age 18 years	Age 19 years	Age 21 years	Age 22 years	Age 23 years
Effector memory CD4^+^ T cells, %	—	—	↑, *85.9* (33.4–74.1)	—	—	—	—	25.3 (6–45)
Effector memory CD8^+^ T cells, %	—	—	52.7 (6.6–76)	—	—	—	—	27.4 (10–62)
Senescent CD4^+^ T cells, %	—	—	0.7 (0.0–0.8)	—	—	—	—	0.2 (0–12)
Senescent CD8^+^ T cells, %	—	—	↑, *22.7* (0–10.3)	—	—	—	—	13.9 (0–66)
CD4:CD8 T-cell ratio	1.5 (0.9–3.4)	1.9 (0.9–3.4)	2 (0.9–3.4)	–	–	↓, *0.6* (1–3.5)	–	1.11 (1–3.5)
Naïve B cells, %	—	—	—	—	—	—	↑, *95.6* (65.6–79.6)	↑, *94.5* (65.6–79.6)
Transitional B cells, %	—	—	—	—	—	—	↑, *15.7* (3.0–5.9)	↑, *14.3* (3.0–5.9)
Plasmablasts, %	—	—	—	—	—	—	1.6 (0.6–1.6)	0.9 (0.6–1.6)
Switched memory B cells, %	—	—	—	—	—	—	↓, *1.9* (7.2–12.7)	↓, *2.9* (7.2–12.7)
Non-switched memory B cells, %	—	—	—	—	—	—	↓, *0.7* (7.4–13.9)	↓, *1.1* (7.4–13.9)
IgG, mg/dl	1,170^a^ (600–1,600)	—	—	1,500^a^ (600–1,600)	630^a^ (600–1,600)	—	860^a^ (600–1,600)	—
IgA, mg/dl	↓, *<7* (80–300)	—	—	↓, *<7* (80–300)	↓, *<7* (80–300)	—	182 (80–300)	—
IgM, mg/dl	↓, *10* (30–250)	—	—	↓, *<10* (30–250)	142 (30–250)	—	35 (30–250)	—

↑, above normal limits; ↓, below normal limits.

^a^ Patient was receiving immunoglobulin replacement therapy.

Normal range values are shown in parentheses. Values outside the normal range are italicized.

Due to CVID, ALPS, recalcitrant ITP, lymphoproliferation, asthma, infections, and a family history of lymphoproliferation, asthma, and infections, genetic testing was conducted at 14 years of age, revealing APDS1 (*PIK3CD* p.Asn334Lys).

Subsequently, he was administered sirolimus (trough concentration goal, 10–15 ng/ml), along with antifungal and antiviral prophylaxes at 14 years of age. Sirolimus improved lymphadenopathy. Lymphocyte subsets were monitored over time, revealing elevated effector memory CD4^+^ and senescent CD8^+^ T cells at 15 years of age. Effector memory CD8^+^ and senescent CD4^+^ T cells were WNLs. A liver biopsy performed at 16 years of age due to splenomegaly and cytopenias revealed mild portal hypertension and perisinusoidal fibrosis. Due to refractory cytopenias, lymphoproliferation, treatment resistance, and progressive multiorgan involvement, P2 underwent a bone marrow transplant at 18 years of age, which resolved the anemia and improved the ITP. Splenomegaly and ITP persisted for several years, resulting in a splenectomy at 21 years of age, which resolved ITP and low WBC, ANC, and ALC.

At 22 years of age, P2 discontinued IVIG, trimethoprim/sulfamethoxazole, and antifungal medications. Naïve and transitional B cells were elevated, while switched and non-switched memory B cells were low. Plasmablasts, IgA, IgM, and IgG were WNLs, and platelet levels were elevated. At 23 years of age, Hgb, WBC, ANC, and ALC were WNLs. Naïve and transitional B cells remained elevated, while switched and non-switched memory B cells remained low. The CD4:CD8 T-cell ratio and effector memory CD4^+^ and senescent CD8^+^ T cells were WNLs.

P2 initially visited the immunology clinic monthly, then every 3 months, and now every 6 months. He also visits his bone marrow transplant team every 9–12 months. He is monitored by neurology for tremors and seizures and is treated with levetiracetam (750 mg twice daily). Due to P2's diagnosis, his half-siblings underwent genetic testing, which identified the same *PIK3CD* variant. As a result, they were diagnosed with APDS early in life and immediately administered immunosuppressive treatment. Both half-siblings have not shown early hematologic manifestations, which we suspect resulted from early diagnosis and treatment.

P2 adhered to and tolerated treatments well with no adverse events.

### Patient 3

Patient 3 (P3) is a 5-year-old Black boy who was referred to a pediatric hematologist/oncologist by his pediatrician at 3 years of age after severe anemia and mild thrombocytopenia were found during admission for an acute GI infection.

His acute GI illness was characterized by hematochezia, abdominal pain, vomiting, and a progressive fever spanning 6 days. During his initial workup, respiratory adenovirus/rhinovirus, *Escherichia coli* urinary tract infection, and *E. coli* in the stool were found. *Clostridioides difficile* toxin and Rocky Mountain spotted fever IgG were positive. Hgb (4.0 g/dl; normal range, 11.5–14.0 g/dl) and platelets (100 × 10^9^/L; normal range, 190–490 × 10^9^/L) were low (Figures 1G,H). WBC (19.64 × 10^9^/L; normal range, 5.6–17 × 10^9^/L) and ANC (9,600 cells/µl; normal range, 900–5,400 cells/µl) were elevated, while ALC was WNLs (Supplementary Figure S2). Hemolytic anemia was excluded, as lactate dehydrogenase, haptoglobin, and Coombs results were WNLs.

Due to anemia and thrombocytopenia, a consultation with pediatric hematology/oncology was requested for evaluation andmanagement. Hematology tests were notable for low iron (22 µg/dl) and transferrin (<80 mg/dl) but high soluble transferrin receptor levels (38 nmol/L). Ferritin levels were normal (24 ng/ml). We suspected that systemic inflammation and iron deficiency (due to blood loss) contributed to his anemia, which was treated with intravenous iron. His GI infection was treated with ciprofloxacin.

During inpatient admission, imaging and laboratory tests were completed to assess nutritional deficiencies, inflammatory conditions, infections, and autoimmune disorders. Vitamin D levels were low, and a skeletal survey indicated mild osteopenia. Parvovirus B19 IgM and IgG were negative, excluding it as a cause of the anemia. Elevated C-reactive protein (135.2 mg/dl) improved but did not normalize after the resolution of P3's acute GI infection. He was positive for cytomegalovirus (CMV) IgM^+^ and IgG^+^ with negative polymerase chain reaction results and positive Epstein–Barr virus nuclear antibody IgG^+^, both indicative of a previous infection. Although an underlying autoimmune disorder could not be excluded completely, it was unlikely since antinuclear antibodies were negative.

Physical examination revealed abdominal tenderness, splenomegaly, and prominent lymphadenopathy overlying the cervical and parotid chains. Concern for peritonitis prompted an abdominal computed tomography scan, which revealed diffuse dilation and a thickened lining of the colon, consistent with enterocolitis, possibly of infectious origin. Tissue transglutaminase IgA testing was negative, excluding celiac disease. Once P3's acute GI infection resolved, his anemia workup was continued at an outpatient pediatric hematology clinic. Hgb increased in response to iron repletion but did not normalize. Cervical lymphadenopathy and splenomegaly persisted.

Several aspects of his presentation, workup, and history raised suspicion for an underlying chronic condition. His medical history revealed recurrent acute otitis media infections from a young age, prompting an adenoidectomy at 2 years of age. Despite the procedure, P2 continued to experience approximately four to six acute otitis media infections per year. He also experienced chronic bloating and recurrent diarrhea. P3's father had a history of lymphadenopathy, upper respiratory infections, and similar GI manifestations throughout childhood and was diagnosed with Crohn's disease. As a young adult, his father required a liver transplant due to alcohol-induced cirrhosis.

A history of recurrent infections and persistent lymphoid hyperplasia raised suspicion for ALPS, prompting immune profiling (Table 3), which revealed low CD4^+^ T cells, CD45RA^+^ naïve T cells, and a CD4:CD8 T-cell ratio. CD8^+^ and CD45RO^+^ memory T cells were elevated. Double-negative T cells were initially elevated (12%) in the setting of a recent multimicrobial infection, but normalized upon re-evaluation, ruling out ALPS. Assessment for underlying immunodeficiency showed normal IgG and IgA but elevated IgM. Tests for human immunodeficiency virus and tuberculosis were negative.

**Table 3:** Immune profiling of patient 3.

Immune subset/immunoglobulin	Age 3 years^a^	Age 3 years 6 months^a^	Age 3 years 7 months^a^	Age 4 years
CD4^+^ T cells, %	—	↓, *12* (27–51)	↓, *18* (25–66)	—
CD8^+^ T cells, %	—	↑, *60* (12–34)	↑, *56* (9–49)	—
CD45RA^+^ naïve T cells, %	—	—	↓, *13* (53–96)	—
CD45RO^+^ memory T cells, %	—	—	↑, *87* (11–50)	—
CD4:CD8 T-cell ratio	—	↓, *0.20* (0.90–2.90)	↓, *0.32* (0.90–2.90)	—
IgG, mg/dl	1,994 (528–2,190)	—	—	2,113^b^ (528–2,190)
IgA, mg/dl	129 (19–220)	—	—	—
IgM, mg/dl	↑, *215* (43–163)	—	—	—

↑, above normal limits; ↓, below normal limits.

^a^ Prior to APDS diagnosis.

^b^ Patient was receiving immunoglobulin replacement therapy.

Normal range values are shown in parentheses. Values outside the normal range are italicized.

Persistent lymphadenopathy, splenomegaly, and anemia raised concerns about leukemia or lymphoma. A chest x-ray showed bilateral peribronchial thickening but no mediastinal mass. Cervical ultrasound showed signs of parotid lymphoid hyperplasia, raising concerns about parotitis. A negative BMBX excluded leukemia. Additional follow-up ultrasounds showed persistent splenomegaly, mild hepatomegaly, thickening of the transverse colon, a distended rectum, and prominent hilar lymph nodes, excluding lymph node abscess.

After excluding several differential diagnoses, persistent anemia, colitis, infections, lymphoproliferation, and a family history of autoimmunity (Crohn's disease) prompted genetic testing at 3 years of age. Results were negative for ALPS and chronic granulomatous disease but revealed a pathogenic variant in *PIK3CD* (p.Glu1021Lys), confirming an APDS1 diagnosis. Subsequently, P3 received sirolimus (trough concentration goal, 5–10 ng/ml) and subcutaneous immunoglobulin treatment (SCIG; 2 g once weekly).

Following the APDS1 diagnosis, an esophagogastroduodenoscopy/colonoscopy revealed colitis and lymphoid aggregates in the GI tract. One year later, an abdominal ultrasound and a chest x-ray were performed to monitor for splenomegaly and assess for bronchiectasis due to a chronic cough, respectively. Chest x-ray results were normal. Treatment with sirolimus and SCIG reduced spleen size (865–313 ml). This dual treatment also resolved thrombocytopenia and anemia, with platelets (233 × 10^9^/L; normal range, 190–491 × 10^9^/L) and Hgb (12.7 g/dl; normal range, 11.5–14.0 g/dl) reaching normal limits at 4 and 5 years of age, respectively. WBC normalized (7.64 × 10^9^/L; normal range, 4.9–12.9 × 10^9^/L) by 4 years of age. P3 received famotidine (8 mg twice daily) for chronic esophagitis, gastritis, and colitis. At 5 years of age, additional imaging of the head, neck, and chest was conducted due to persistent and severe lymphadenopathy. The results revealed bilateral submandibular lymphadenopathy, persistent swollen parotid glands, and bilateral lower lobe atelectasis.

P3 initially visited hematology/oncology every 2 weeks. Now, at 5 years of age, these visits have been reduced to every 4–6 weeks. Follow-ups with infectious disease, endocrinology, otolaryngology, and gastroenterology are scheduled as needed. He visits immunology every 6 months. Since receiving sirolimus, he had no recurrence of GI symptoms or severe infections, nor has he required hospitalization. Intermittent severe and prolonged lymphadenopathy, parotitis, and mild infections (viral upper respiratory, acute otitis media, and lymphadenitis) persist.

P3's diagnosis resulted in the relatives re-evaluating their medical histories. P3's father had undergone extensive workup as a child and was seen by multiple specialists, with no unifying diagnosis. Genetic testing of P3's father identified the same pathogenic *PIK3CD* variant.

SCIG treatment remains challenging for P3 and his family, but they are satisfied with the resolution of his GI manifestations and reduced infection frequency. P3's family is eager to see a reduction in his persistent lymphadenopathy. No adverse events have been reported during P3's treatment.

## Discussion

These cases describe the diagnostic journey and management of three patients with APDS who presented to a pediatric hematologist/oncologist with early-onset and/or recalcitrant cytopenias. Genetic evaluation was crucial for reaching an APDS diagnosis, which led to the administration of targeted therapeutics and improved outcomes. These cases underscore the importance of considering IEIs in patients with early-onset, chronic, and/or recalcitrant cytopenias.

Although hematologic manifestations have been reported in several IEIs, they can be challenging to recognize due to the lack of specific biomarkers to identify such patients. Nevertheless, hematologic warning signs for IEIs have been published and may include features such as persistent, early, or multilineage cytopenias and hepatosplenomegaly (19, 20). Therefore, patients with hematologic warning signs should be screened for an underlying IEI, as these manifestations may be strong indicators of these rare diseases (21).

Supporting this notion, underlying IEIs have been reported in 40%–67% of pediatric patients with Evans syndrome or multilineage cytopenias (22–24). Similarly, an estimated 15% of pediatric autoimmune anemia cases in France may be linked to an underlying IEI (25). Cytopenias are not the only manifestation of IEIs that hematologists/oncologists may encounter. In APDS, 71%–89% of patients have benign lymphoproliferation, and 13%–28% develop malignancy, particularly lymphoma (4, 10, 11, 13, 26). Collectively, hematologists/oncologists are likely to manage patients with IEIs, suggesting that early genetic testing should be at the forefront of the diagnostic workup for patients with early-onset and/or refractory cytopenias. Identifying an underlying genetic variant can provide insight into disease pathophysiology and impact treatment decisions (27). For example, following their APDS diagnosis, cytopenias improved in two patients who received modified treatment that included IVIG/SCIG, bone marrow transplant, splenectomy, or sirolimus. The latter has previously been administered to patients with APDS as it acts downstream of PI3Kδ to inhibit the mammalian target of rapamycin (4, 28). There is an FDA-approved PI3Kδ inhibitor (leniolisib) for the treatment of APDS in patients aged ≥12 years, but none of the patients described received this form of treatment, as two were underage and the third is doing well post-BMT (29). Questions remain regarding the use of leniolisib as a bridge to hematopoietic stem cell transplant. Risks such as graft failure should be considered when determining the relevance of using leniolisib as a bridge to transplant (30, 31). Collectively, these cases underscore the importance of initiating early genetic testing, which can provide diagnostic information, uncover targeted therapeutics, and improve outcomes.

Establishing an early genetic diagnosis in a patient may prompt genetic testing in relatives, as evidenced by the additional family members who were diagnosed with APDS. Genetic testing is particularly important in families because APDS has an autosomal dominant inheritance pattern (8). While all patients were diagnosed with APDS1, two patients had a pathogenic E1021K variant identified in *PIK3CD*, which is the most commonly reported variant in patients with APDS1 (32). Family testing allowed early diagnosis and treatment of P3's half-siblings before developing hematologic complications. These cases highlight the benefits of genetic testing for relatives of patients with APDS.

These cases have influenced our approach to complex hematologic manifestations. We now approach unusual cases by reviewing the medical history, initiating an immune workup, utilizing genetic testing panels that screen for cytopenias and IEIs, collaborating with immunology, and referring patients earlier in the diagnostic process. We find that suspicion of an underlying IEI is warranted in cases of early-onset or refractory cytopenias. Although this report is limited to three cases, the work of Rotz et al. also supports genetic testing by hematologists/oncologists in patients with unusual disease characteristics such as multilineage cytopenias and cytopenias in the presence of lymphoproliferation or autoimmune disease (1).

Currently, there are few case reports describing the diagnostic journey of patients with APDS who present with early-onset or recurrent cytopenias (33, 34). While previous case reports have focused on either treatment outcomes with hematopoietic stem cell transplant (HSCT) or autoimmune hemolytic anemia in the setting of APDS, this case series highlights the variety of cytopenias that patients with APDS may exhibit, including early-onset ITP, severe anemia, and/or multilineage cytopenias.

We hope that this case series will encourage other hematologists/oncologists to consider IEIs as a differential diagnosis in complex cases. These cases suggest that underlying IEIs should be considered in the workup of recalcitrant cytopenias, particularly in patients with a combination of hematologic and immunologic manifestations that are refractory to treatment, manifest at an unusually young age, or can be tied to family history.

## Data Availability

The datasets presented in this article are not readily available because of ethical and privacy restrictions. Requests to access the datasets should be directed to the corresponding author.

## References

[1] Rotz SJ Ware RE Kumar A. Diagnosis and management of chronic and refractory immune cytopenias in children, adolescents, and young adults. Pediatr Blood Cancer. (2018) 65(10):e27260. 10.1002/pbc.27260 29856527

[2] Teachey DT Lambert MP. Diagnosis and management of autoimmune cytopenias in childhood. Pediatr Clin North Am. (2013) 60(6):1489–511. 10.1016/j.pcl.2013.08.009 24237984 PMC5384653

[3] Lougaris V Piane FL Cancrini C Conti F Tommasini A Badolato R Activated phosphoinositde 3-kinase (PI3Kdelta) syndrome: an Italian point of view on diagnosis and new advances in treatment. Ital J Pediatr. (2024) 50(1):103. 10.1186/s13052-024-01662-5 38769568 PMC11106885

[4] Maccari ME Abolhassani H Aghamohammadi A Aiuti A Aleinikova O Bangs C Disease evolution and response to rapamycin in activated phosphoinositide 3-kinase delta syndrome: the European society for immunodeficiencies-activated phosphoinositide 3-kinase delta syndrome registry. Front Immunol. (2018) 9:543. 10.3389/fimmu.2018.00543 29599784 PMC5863269

[5] Michalovich D Nejentsev S. Activated PI3 kinase delta syndrome: from genetics to therapy. Front Immunol. (2018) 9:369. 10.3389/fimmu.2018.00369 29535736 PMC5835040

[6] Redenbaugh V Coulter T. Disorders related to PI3Kdelta hyperactivation: characterizing the clinical and immunological features of activated PI3-kinase delta syndromes. Front Pediatr. (2021) 9:702872. 10.3389/fped.2021.702872 34422726 PMC8374435

[7] Singh A Joshi V Jindal AK Mathew B Rawat A. An updated review on activated PI3 kinase delta syndrome (APDS). Genes Dis. (2020) 7(1):67–74. 10.1016/j.gendis.2019.09.015 32181277 PMC7063426

[8] Lucas CL Kuehn HS Zhao F Niemela JE Deenick EK Palendira U Dominant-activating germline mutations in the gene encoding the PI(3)K catalytic subunit p110delta result in T cell senescence and human immunodeficiency. Nat Immunol. (2014) 15(1):88–97. 10.1038/ni.2771 24165795 PMC4209962

[9] Lucas CL Zhang Y Venida A Wang Y Hughes J McElwee J Heterozygous splice mutation in PIK3R1 causes human immunodeficiency with lymphoproliferation due to dominant activation of PI3K. J Exp Med. (2014) 211(13):2537–47. 10.1084/jem.20141759 25488983 PMC4267241

[10] Coulter TI Chandra A Bacon CM Babar J Curtis J Screaton N Clinical spectrum and features of activated phosphoinositide 3-kinase delta syndrome: a large patient cohort study. J Allergy Clin Immunol. (2017) 139(2):597–606.e4. 10.1016/j.jaci.2016.06.021 27555459 PMC5292996

[11] Elkaim E Neven B Bruneau J Mitsui-Sekinaka K Stanislas A Heurtier L Clinical and immunologic phenotype associated with activated phosphoinositide 3-kinase delta syndrome 2: a cohort study. J Allergy Clin Immunol. (2016) 138(1):210–8.e9. 10.1016/j.jaci.2016.03.022 27221134

[12] Nunes-Santos CJ Uzel G Rosenzweig SD. PI3K pathway defects leading to immunodeficiency and immune dysregulation. J Allergy Clin Immunol. (2019) 143(5):1676–87. 10.1016/j.jaci.2019.03.017 31060715

[13] Jamee M Moniri S Zaki-Dizaji M Olbrich P Yazdani R Jadidi-Niaragh F Clinical, immunological, and genetic features in patients with activated Pi3kdelta syndrome (APDS): a systematic review. Clin Rev Allergy Immunol. (2020) 59(3):323–33. 10.1007/s12016-019-08738-9 31111319

[14] Wang Y Wang W Liu L Hou J Ying W Hui X Report of a Chinese cohort with activated phosphoinositide 3-kinase delta syndrome. J Clin Immunol. (2018) 38(8):854–63. 10.1007/s10875-018-0568-x 30499059

[15] Baleydier F Ranza E Schappi M Rougemont AL Merlini L Ansari M Activated phosphoinositide 3 kinase delta syndrome (APDS): a primary immunodeficiency mimicking lymphoma. J Pediatr Hematol Oncol. (2019) 41(8):e521–4. 10.1097/MPH.0000000000001328 30334905

[16] Conti F Catelli A Cifaldi C Leonardi L Mule R Fusconi M Case report: Hodgkin lymphoma and refractory systemic lupus erythematosus unveil activated phosphoinositide 3-kinase-delta syndrome 2 in an adult patient. Front Pediatr. (2021) 9:702546. 10.3389/fped.2021.702546 34307262 PMC8295470

[17] Rivalta B Amodio D Milito C Chiriaco M Di Cesare S Giancotta C Case report: EBV chronic infection and lymphoproliferation in four APDS patients: the challenge of proper characterization, therapy, and follow-up. Front Pediatr. (2021) 9:703853. 10.3389/fped.2021.703853 34540765 PMC8448282

[18] Yin Z Tian X Zou R He X Chen K Zhu C. Case report: first occurrence of plasmablastic lymphoma in activated phosphoinositide 3-kinase delta syndrome. Front Immunol. (2021) 12:813261. 10.3389/fimmu.2021.813261 34992612 PMC8724197

[19] Costagliola G Peroni DG Consolini R. Beyond infections: new warning signs for inborn errors of immunity in children. Front Pediatr. (2022) 10:855445. 10.3389/fped.2022.855445 35757131 PMC9226481

[20] Sanchez-Ramon S Bermudez A Gonzalez-Granado LI Rodriguez-Gallego C Sastre A Soler-Palacin P Primary and secondary immunodeficiency diseases in oncohaematology: warning signs, diagnosis, and management. Front Immunol. (2019) 10:586. 10.3389/fimmu.2019.00586 30984175 PMC6448689

[21] Dabrowska A Grzesk E Urbanczyk A Mazalon M Grzesk G Styczynski J Extended list of warning signs in qualification to diagnosis and treatment of inborn errors of immunity in children and young adults. J Clin Med. (2023) 12(10):3401. 10.3390/jcm12103401 37240507 PMC10219467

[22] Hadjadj J Aladjidi N Fernandes H Leverger G Magerus-Chatinet A Mazerolles F Pediatric Evans syndrome is associated with a high frequency of potentially damaging variants in immune genes. Blood. (2019) 134(1):9–21. 10.1182/blood-2018-11-887141 30940614

[23] Miano M Guardo D Grossi A Palmisani E Fioredda F Terranova P Underlying inborn errors of immunity in patients with Evans syndrome and multilineage cytopenias: a single-centre analysis. Front Immunol. (2022) 13:869033. 10.3389/fimmu.2022.869033 35655776 PMC9152001

[24] Rivalta B Zama D Pancaldi G Facchini E Cantarini ME Miniaci A Evans syndrome in childhood: long term follow-up and the evolution in primary immunodeficiency or rheumatological disease. Front Pediatr. (2019) 7:304. 10.3389/fped.2019.00304 31396497 PMC6664023

[25] Fischer A Provot J Jais JP Alcais A. Mahlaoui N, Members of the CEREDIH French PID study group. Autoimmune and inflammatory manifestations occur frequently in patients with primary immunodeficiencies. J Allergy Clin Immunol. (2017) 140(5):1388–93.e8. 10.1016/j.jaci.2016.12.978 28192146

[26] Carpier JM Lucas CL. Epstein-Barr virus susceptibility in activated PI3Kdelta syndrome (APDS) immunodeficiency. Front Immunol. (2017) 8:2005. 10.3389/fimmu.2017.02005 29387064 PMC5776011

[27] Quinn J Modell V Johnson B Poll S Aradhya S Orange JS Global expansion of Jeffrey’s insights: Jeffrey Modell Foundation’s genetic sequencing program for primary immunodeficiency. Front Immunol. (2022) 13:906540. 10.3389/fimmu.2022.906540 35757720 PMC9226364

[28] Berglund LJ. Modulating the PI3K signalling pathway in activated PI3K delta syndrome: a clinical perspective. J Clin Immunol. (2023) 44(1):34. 10.1007/s10875-023-01626-0 38148368 PMC10751257

[29] Joenja [Package Insert]. Leiden, the Netherlands. Pharming Technologies B.V. (2023).

[30] Albert MH Sirait T Eikema DJ Bakunina K Wehr C Suarez F Hematopoietic stem cell transplantation for adolescents and adults with inborn errors of immunity: an EBMT IEWP study. Blood. (2022) 140(14):1635–49. 10.1182/blood.2022015506 35344580

[31] Dimitrova D Nademi Z Maccari ME Ehl S Uzel G Tomoda T International retrospective study of allogeneic hematopoietic cell transplantation for activated PI3K-delta syndrome. J Allergy Clin Immunol. (2022) 149(1):410–21.e7. 10.1016/j.jaci.2021.04.036 34033842 PMC8611111

[32] Maccari ME Wolkewitz M Schwab C Lorenzini T Leiding JW Aladjdi N Activated phosphoinositide 3-kinase delta syndrome: update from the ESID registry and comparison with other autoimmune-lymphoproliferative inborn errors of immunity. J Allergy Clin Immunol. (2023) 152(4):984–96.e10. 10.1016/j.jaci.2023.06.015 37390899

[33] Schworer SA Francis OL Johnson SM Smith BD Gold SH Smitherman AB Autoimmune cytopenia as an early and initial presenting manifestation in activated PI3 kinase delta syndrome: case report and review. J Pediatr Hematol Oncol. (2021) 43(8):281–7. 10.1097/MPH.0000000000002214 34054047 PMC8542580

[34] Yang X Xi R Bai J Pan Y. Successful haploidentical hematopoietic stem cell transplantation for activated phosphoinositide 3-kinase delta syndrome: case report and literature review. Medicine (Baltimore). (2023) 102(5):e32816. 10.1097/MD.0000000000032816 36749229 PMC9902017

[35] van Gent R van Tilburg CM Nibbelke EE Otto SA Gaiser JF Janssens-Korpela PL Refined characterization and reference values of the pediatric T- and B-cell compartments. Clin Immunol. (2009) 133(1):95–107. 10.1016/j.clim.2009.05.020 19586803

[36] Morbach H Eichhorn EM Liese JG Girschick HJ. Reference values for B cell subpopulations from infancy to adulthood. Clin Exp Immunol. (2010) 162(2):271–9. 10.1111/j.1365-2249.2010.04206.x 20854328 PMC2996594

