# Supplementary material for: Case Report: Early-onset or recalcitrant cytopenias as presenting manifestations of activated PI3Kδ syndrome
Source: Front Pediatr. 2024 Nov 27;12:1494945. doi: 10.3389/fped.2024.1494945 (PMC11632462; doi:10.3389/fped.2024.1494945)
Supplement: Supplementary file 1 [file Datasheet1.pdf]

CARE
case report guidelines

CARE Checklist of information to include when writing a case report

(cc) BY-NC-ND

Topic	Item	Checklist item description	Reported on Line
**Title**	1	The diagnosis or intervention of primary focus followed by the words "case report"	1-2
**Key Words**	2	2 to 5 key words that identify diagnoses or interventions in this case report, including "case report"	15-16
**Abstract (no references)**	3a	Introduction: What is unique about this case and what does it add to the scientific literature?	18-24
	3b	Main symptoms and/or important clinical findings	25-31
	3c	The main diagnoses, therapeutic interventions, and outcomes	31-37
	3d	Conclusion—What is the main "take-away" lesson(s) from this case?	38-44
**Introduction**	4	One or two paragraphs summarizing why this case is unique (**may include references**)	47-60
**Patient Information**	5a	De-identified patient specific information.	P1: 63 P2: 103 P3: 164
	5b	Primary concerns and symptoms of the patient.	P1: 64 P2: 104 P3:165
	5c	Medical, family, and psycho-social history including relevant genetic information	P1: 74-77 P2: 106-110 P3: 194-198
	5d	Relevant past interventions with outcomes	P1: 67-69, 74 P2: 107-109 P3: 176-178
**Clinical Findings**	6	Describe significant physical examination (PE) and important clinical findings.	P1: 65-74 P2: 105-106 P3: 167-187
**Timeline**	7	Historical and current information from this episode of care organized as a timeline	Figure 1A
**Diagnostic Assessment**	8a	Diagnostic testing (such as PE, laboratory testing, imaging, surveys).	P1: 73-80 P2: 116-119, 121-112, 129-135 P3: 187-191, 199-209
	8b	Diagnostic challenges (such as access to testing, financial, or cultural)	N/A but challenges reaching accurate rare disease diagnosis are reported above
	8c	Diagnosis (including other diagnoses considered)	P1: 80-81 P2: 136-138 P3: 212-213
	8d	Prognosis (such as staging in oncology) where applicable	P1:87-88 P2: 155-157, 160-161 P3: 228-233
**Therapeutic Intervention**	9a	Types of therapeutic intervention (such as pharmacologic, surgical, preventive, self-care)	P1: 67-69, 94-95 P2: 114-115, 139-140, 156-1157 P3: 176-178, 213-215, 223-224
	9b	Administration of therapeutic intervention (such as dosage, strength, duration)	P1: 67-69 P2: 139, 156-157 P3: 213-214, 223-224
	9c	Changes in therapeutic intervention (with rationale)	P1: 88 P2: 144-148, 149, 155-156 P3: 228-230
**Follow-up and Outcomes**	10a	Clinician and patient-assessed outcomes (if available)	P3: 230-232, 237-240
	10b	Important follow-up diagnostic and other test results	P1: 82-83, 88-89, 98-100 P2: 140-142, 145-147, 149-154, 157-158 P3: 216-227, 234-236
	10c	Intervention adherence and tolerability (How was this assessed?)	P1: 69 P2:162 P3: 237
	10d	Adverse and unanticipated events	P1: 101 P2: 162 P3: 239-240
**Discussion**	11a	A scientific discussion of the strengths AND limitations associated with this case report	245-246, 256-260, 265-266, 267-271
	11b	Discussion of the relevant medical literature **with references**.	247-252
	11c	The scientific rationale for any conclusions (including assessment of possible causes)	243-245, 255-258, 261-263, 271-274
	11d	The primary "take-away" lessons of this case report (without references) in a one paragraph conclusion	275-279
**Patient Perspective**	12	The patient should share their perspective in one to two paragraphs on the treatment(s) they received	P3: 237-239
**Informed Consent**	13	Did the patient give informed consent? Please provide if requested	**Yes** ☑ **No** ☐
