# Supplementary material for: Case Report: Early-onset or recalcitrant cytopenias as presenting manifestations of activated PI3Kδ syndrome
Source: Front Pediatr. 2024 Nov 27;12:1494945. doi: 10.3389/fped.2024.1494945 (PMC11632462; doi:10.3389/fped.2024.1494945)
Supplement: Supplementary file 2 [file Datasheet2.docx]

**Supplementary Materials.**


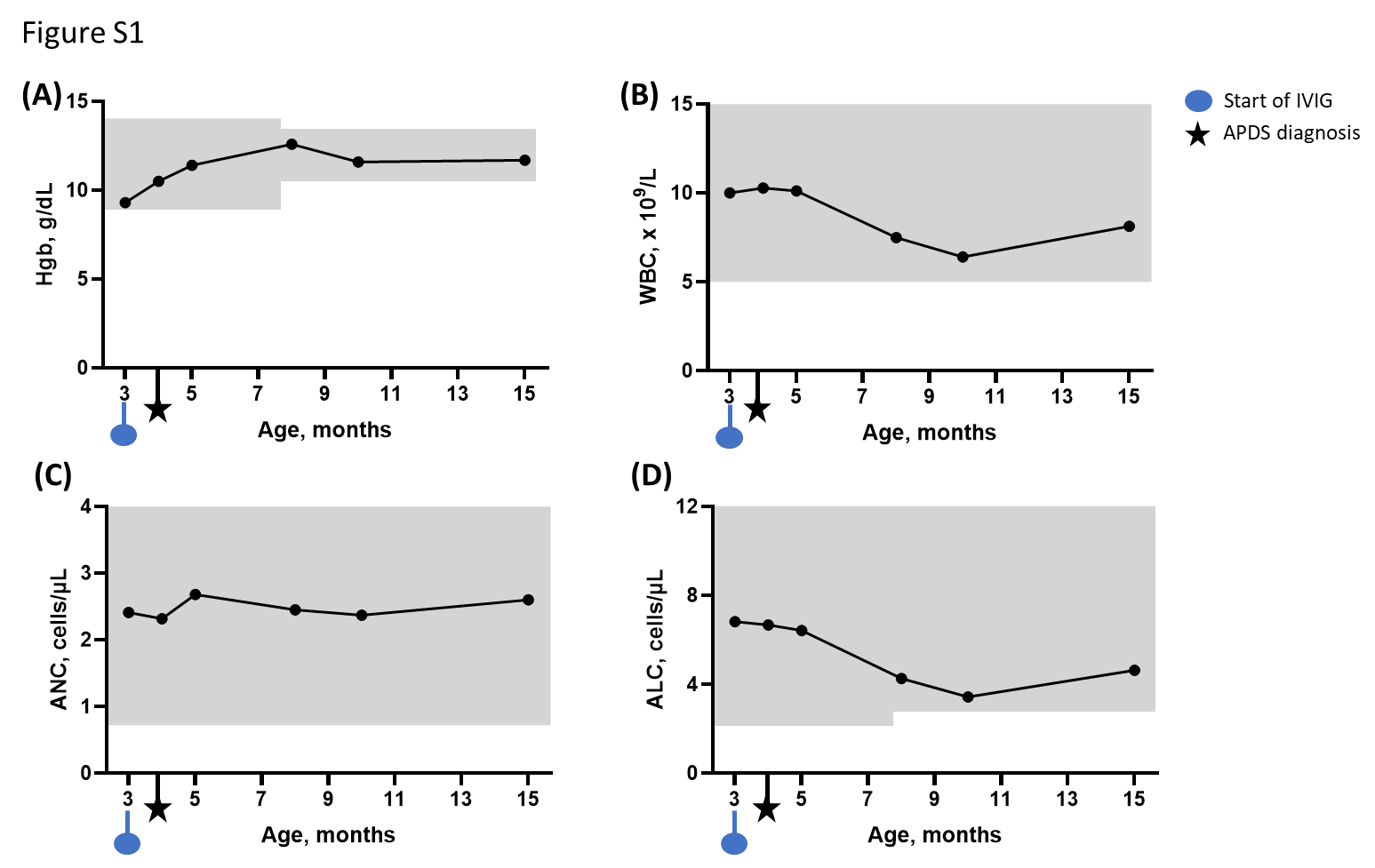


**Figure S1. Patient 1 Hgb, WBC, ANC, and ALC over time.** Gray boxes indicate normal range. Three values were averaged to plot data at 4 months old. ALC, absolute lymphocyte count; ANC, absolute neutrophil count; Hgb, hemoglobin; WBC, white blood cell count.


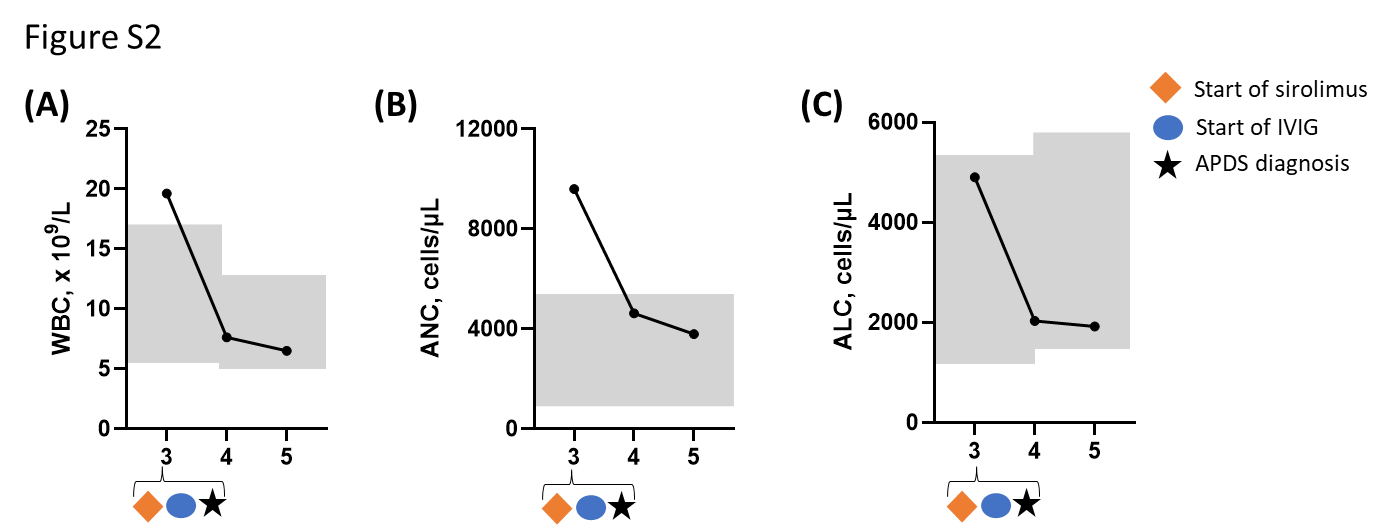


**Figure S2. Patient 3 WBC, ANC, and ALC over time.** Gray boxes indicate normal range(18, 19). ALC, absolute lymphocyte count; ANC, absolute neutrophil count; WBC, white blood cell count.
